# Novel 9-(alkylthio)-Acenaphtho[1,2-e]-1,2,4-triazine derivatives: synthesis, cytotoxic activity and molecular docking studies on B-cell lymphoma 2 (Bcl-2)

**DOI:** 10.1186/2008-2231-22-2

**Published:** 2014-01-06

**Authors:** Mohammad K Mohammadi, Omidreza Firuzi, Mehdi Khoshneviszadeh, Nima Razzaghi-Asl, Saghi Sepehri, Ramin Miri

**Affiliations:** 1 Faculty of sciences, Ahvaz Branch, Islamic Azad University, Ahvaz, Iran; 2 Medicinal and Natural Products Chemistry Research Center, Shiraz, University of Medical Sciences, PO Box 3288–71345, Shiraz, Iran; 3 Departments of Medicinal Chemistry, School of Pharmacy, Shiraz University of Medical Sciences, Shiraz, Iran; 4 Departments of Medicinal Chemistry, Faculty of Pharmacy, Isfahan University of Medical Sciences, Isfahan, Iran

**Keywords:** Synthesis, Acenaphtho-9,10-quinone, Cytotoxic activity, Docking

## Abstract

**Background and purpose of the study:**

Acenaphtho derivatives have been reported as antitumor agents. Due to this fact and also with the aim of developing the chemistry of potentially bioactive heterocyclic compounds via efficient reactions, a facile procedure for the synthesis of 9-(alkylthio)-acenaphtho[1,2-e]-1,2,4-triazines via two step condensation of thiosemicarbazide and acenaphtylene-9,10-quinone to form acenaphtho[1,2-e]-1,2,4-triazine-9(8H)-thiones and subsequent reaction with benzyl chloride derivatives is reported.

**Methods:**

9-(alkylthio) acenaphtho[1,2-e]-1,2,4-triazines were synthesized via the reaction of acenaphtho-9,10-quinone with thiosemicarbazide, and then with the benzyl chloride derivatives. Cytotoxicity of some prepared compounds was assessed through MTT assay on three different human cancerous cell lines (HL-60, MCF7, and MOLT-4 cells). Molecular docking studies were performed via AutoDock4.2 software in order to confirm an apoptosis-inducing activity of acenaphtho scaffolds via the Bcl-2 protein.

**Results:**

Excellent yields of the products, short reaction times and simple work-up are attractive features of this synthetic protocol. The evaluated compounds exhibited moderate to good cytotoxic activities. Docking results on the active site of B-cell lymphoma 2 (Bcl-2) supported the experimental biological data and agreed well with previous *in silico* data for commonly used anti-cancer drugs. Moreover; results were analyzed considering binding efficiency indices.

**Conclusions:**

The outcomes of the present study may be helpful in future targeting of Bcl-2 with the aim of developing apoptosis-inducing agents.

## Introduction

Economic generation of bioactive compounds has been a major concern in modern organic chemistry [1]. In this regard, development of novel compounds and especially diverse small molecule scaffolds caused higher attention of medicinal and biological chemists [2-4]. This can be attributed to the growing requirement in assembling libraries of structurally complex substances to be evaluated as hit/lead compounds in drug discovery projects.

Polycyclic aromatic hydrocarbon (PAH) heterocycles are highly important structural units in a variety of pharmacologically active substances [5-9]. At first glance, rigid polycyclic structures seem to have role in the development of antitumor agents owing to their ability in insertion between stacked base pairs of oligonucleotides and action as intercalator [10-12]. Particularly important is that when these planar polycyclic heterocycles bear appropriate side chains, further interactions with other important macromolecules might be envisaged [11,13].

In this view, privileged heterocyclic structures have been constructed around the acenaphtho core [14,15]. Some of the acenaphtho derivatives containing thiazole backbone have been reported as antitumor agents [16]. Recently in an attempt to develop protein-targeted instead of DNA-targeted antitumor agents, some derivatives of 8H-acenaphtho[1,2-b]pyrrole have been constructed [17].

The authors demonstrated that 8-oxo-3-thiomorpholin-4-yl-8H-acenaphtho[1,2-b]pyrrole-9-carbonitrile could serve as an apoptosis-inducing agent via interacting Bcl-2 protein [17]. It is well known that the Bcl-2 family of proteins is comprised of pro-apoptotic and anti-apoptotic proteins and all members of this family are not anti-apoptotic. Anti-apoptotic Bcl-2 family proteins, including Bcl-2, Bcl-XL, Bcl-w, Mcl-1 and A1, prevent cell death by binding and sequestering pro-apoptotic proteins so, inhibition of these anti-apoptotic proteins might be lethal to cancer cells.

Indeed, Bcl-2 proteins have been regarded as important targets for anti-neoplastic drug development and Bcl-2 gen has been identified as over expressed in various cancers. Bcl-2 is an anti-apoptotic protein possessing an important role in various types of cancers. Bcl-2 is the member of the Bcl-2 family of apoptosis regulator proteins which is encoded by the BCL2 gene [18,19].

Various reactions of acenaphthaquinone with nucleophiles, organic and inorganic reagents have been reviewed elsewhere [20,21]. In the framework of our program to develop the chemistry of potentially bioactive heterocyclic compounds [22] and in connection with our ongoing interests in this field [23-25], we represent here a facile procedure for the synthesis of 9-(alkylthio)-acenaphtho[1,2-e]-1,2,4-triazines via two step condensation of thiosemicarbazide and acenaphtylene-9,10-quinone to form acenaphtho[1,2-e]-1,2,4-triazine-9(8H)-thiones and subsequent reaction with benzyl chloride derivatives. Prepared compounds were subjected to cytotoxic assay in three different cancerous cell lines. Moreover; molecular docking was used to gain further insight into the binding mode and binding affinity of acenaphtho derivatives in the active site of Bcl-2.

## Material and methods

All of the reagents were purchased from commercial sources and were freshly used after being purified by standard procedures. Melting points were determined on the Electro-thermal Melting Point apparatus and were uncorrected. Infrared spectra were recorded on the Shimadzu-420 infrared spectrophotometer. ^1^H-NMR and^13^C-NMR spectra were recorded in DMSO-d_6_ or CDCl_3_ on Brucker 300 MHz spectrometer (Chemical shifts are given in parts per million or ppm). Mass spectra were recorded on a MS model 5973 Network apparatus at ionization potential of 70 eV. Elemental analyses (C, H, N) were performed by the Microanalytical Unit.

### General procedure for preparation of acenaphtho[1,2-e]-1,2,4-triazine-9(8H)-thione (3)

**Scheme 1:**
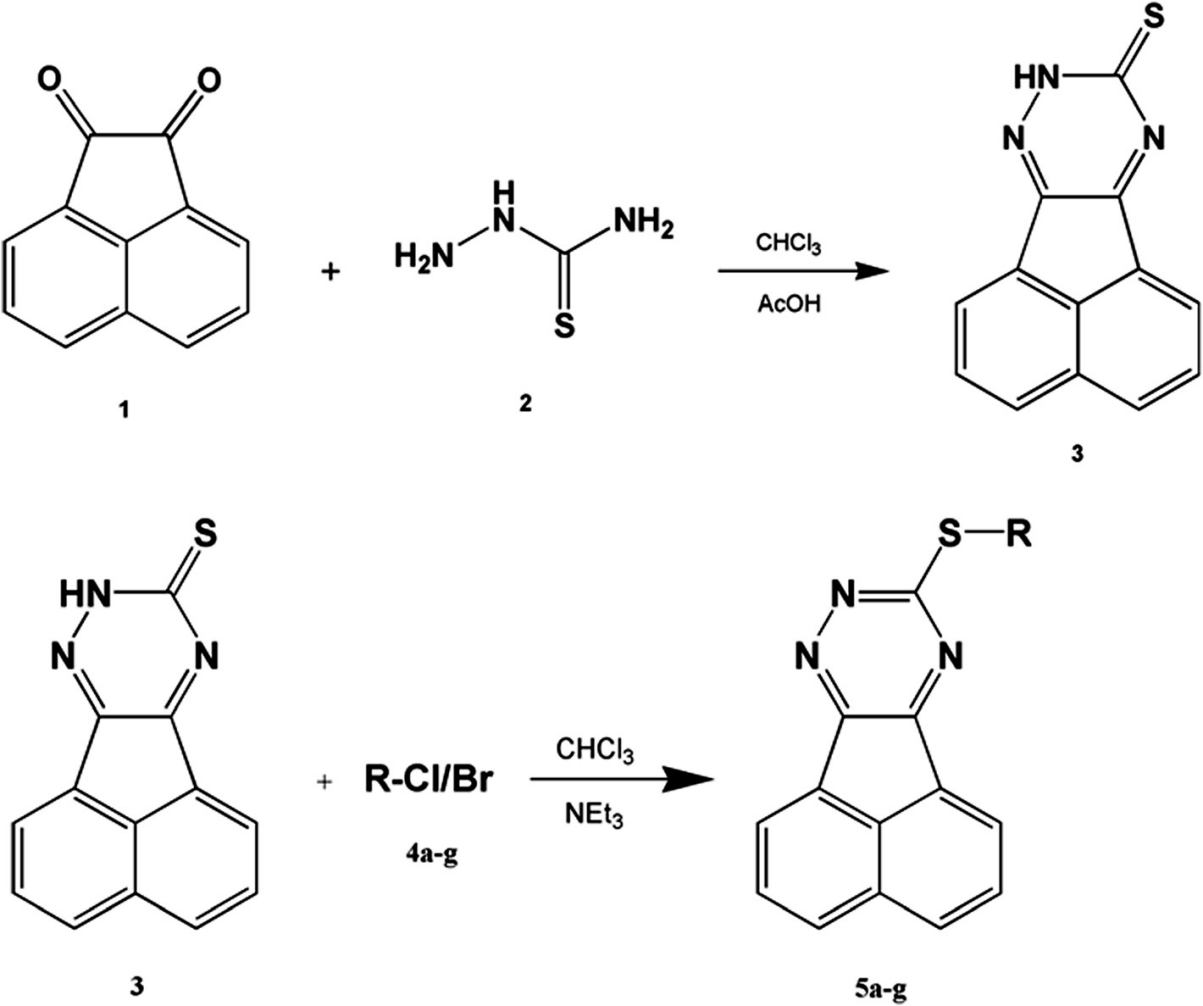
Synthetic rout to acenaphtho[1,2-e]-1,2,4-triazine-9(8H)-thione and 9-(alkylthio)-acenaphtho[1,2-e]-1,2,4-triazines.

To the acenaphtylene-9,10-quinone (5 mmol) and thiosemicarbazide (5 mmol) in chloroform (30 mL), small amount of acetic acid was added as an catalyst. The reaction mixture was stirred under reflux condition. The progress of the reaction was monitored with TLC and at the completion of the reaction, The precipitated product was filtered off, washed with mixture of H_2_O/EtOH, dried and recrystallized from ethanol to give yellow crystalline acenaphtho[1,2-e]-1,2,4-triazine-9(8H)-thione (Scheme 1).

### General procedure for preparation of 9-(alkylthio)-acenaphtho[1,2-e]-1,2,4-triazines (5a-g)

To a well-stirred solution of acenaphtho[1,2-e]-1,2,4-triazine-9(8H)-thione (**3**) in 10 ml Chloroform was added triethylamine (3 mmol).

The solution was stirred and then the benzyl chloride derivatives, methyl iodide or ethyl iodide were added and the mixture was heated and stirred under reflux condition. After completion of the reactions, the precipitated residue was filtered, recrystallized in ethanol, filtered, washed with water (2 × 5 mL) and then completely dried in electrical oven (Scheme 1). All prepared compounds were characterized using FT-IR, ^1^H NMR, ^13^C NMR and mass spectroscopy (Additional file 1: Table S1).

#### ***Acenaphtho[1,2-e]-1,2,4-triazine-9(8H)-thione (3)***

Yield 88%, m.p. 148-150°C. ^1^HNMR (300 MHz, DMSO-d_6_) δ: 7.81 (d, 2H, J = 7.5Hz, CH-aromatic), 7.65 (dd, 2H, J = 7.6, 5.9 Hz, CH-aromatic), 7.46 (d, 2H, J = 8Hz CH-aromatic), 3.21 (s, 1H, SH); IR (KBr, cm^–1^): 3245, 3151, 2922, 1689, 1607, 777; ^13^C-NMR (75 MHz, DMSO-d_6_) δ: 163, 150, 131, 128, 126, 124, 123; MS: m/z (%) 255 (M^+^, 51), 213 (100), 180 (75), 152 (49), 139 (44); Anal. Calcd for C_13_H_7_N_3_S: C, 65.80; H, 2.97; N, 17.71. Found: C, 65.62; H, 2.98; N, 17.58.

#### ***9-(benzylthio)-acenaphtho[1,2-e]-1,2,4-triazine (5a)***

Yield 94%, m.p. 151-153°C. ^1^HNMR (300 MHz, DMSO-d_6_) δ: 7.78 (d, 2H, J = 7.6Hz, CH-aromatic), 7.61 (dd, 2H, J = 7.6, 6.6 Hz, CH-aromatic), 7.46 (d, 2H, J = 8.5Hz, CH-aromatic), 7.05-7.19 (m, 5H, CH-phenyl), 3.26 (s, 2H, CH_2_-benzylic); IR (KBr, cm^–1^): 3153, 3050, 1694, 1606; ^13^C-NMR (75 MHz, DMSO-d6) δ: 170.9, 139.7, 142.4, 138.9, 133.5, 128.5, 128.3, 128, 127.8, 127.7, 127.3, 127.2, 126.6, 124.5, 42.2; MS: m/z (%) 327 (M^+^, 100), 294 (63), 208 (71), 164 (52), 91 (75); Anal. Calcd for C_20_H_13_N_3_S: C, 73.37; H, 4.00; N, 12.83. Found: C, 73.62; H, 3.88; N, 12.58.

#### ***9-(4-nitro-benzylthio)-acenaphtho[1,2-e]-1,2,4-triazine (5b)***

Yield 79%, m.p. 156-158°C. ^1^HNMR (300 MHz, DMSO-d_6_) δ: 8.08 (d, 2H, J = 7.5Hz, CH-phenyl), 7.83 (d, 2H, J = 7.6Hz, CH-aromatic), 7.75 (dd, 2H, J = 7.3, 6.6 Hz, CH-aromatic), 7.46 (d, 2H, J = 7.6Hz, CH-phenyl), 7.48 (d, 2H, CH-phenyl), 3.32 (s, 2H, CH_2_-benzylic); IR (KBr, cm^–1^): 3121, 3035, 1670, 1585, 1532, 1486, 937; ^13^C-NMR (75 MHz, DMSO-d6) δ: 172.4, 149.8, 145.8, 142.4, 133.5, 131.7, 130.3, 127.6, 127.8, 127.3, 128.9, 128.5, 127.5, 124.1, 44.5; MS: m/z (%) 372 (M^+^, 12), 255 (48), 213 (100), 180 (87), 152 (62); Anal. Calcd for C_20_H_12_N_4_O_2_S: C, 64.50; H, 3.25; N, 15.04. Found: C, 64.62; H, 3.18; N, 15.28.

#### ***9-(2,4-dichloro-benzylthio)-acenaphtho[1,2-e]-1,2,4-triazine (5c)***

Yield 83%, m.p. 187-188°C. ^1^HNMR (300 MHz, DMSO-d_6_) δ: 7.79 (d, 2H, J = 7.5Hz, CH-aromatic), 7.60 (dd, 2H, J = 7.5, 6.1 Hz, CH-aromatic), 7.46 (d, 2H, J = 8.3Hz CH-aromatic), 7.19 (s, 1H, CH-phenyl), 7.09 (d, 1H, J = 7.3Hz, CH-phenyl), 6.98 (d, 1H, J = 7.3Hz, CH-phenyl), 3.33 (s, 2H, CH_2_-benzylic); IR (KBr, cm^–1^): 3175, 3095, 1656, 1578, 951, 875, 839; ^13^C-NMR (75 MHz, DMSO-d6) δ: 171.8, 145.4, 134.5, 134.1, 133.5, 131.4, 130.8, 130.4, 129.2, 128.6, 128.1, 127.9, 127.6, 127.3, 127.1, 125.6, 40.4; MS: m/z (%) 395 (M^+^, 29), 360 (100), 208 (94), 164 (58); Anal. Calcd for C_20_H_11_Cl_2_N_3_S: C, 60.62; H, 2.80; N, 10.60. Found: C, 60.42; H, 2.91; N, 10.73.

#### ***9-(3,4-dichloro -benzylthio)-acenaphtho[1,2-e]-1,2,4-triazine (5d)***

Yield 60%, m.p. 156-159°C. ^1^HNMR (300 MHz, DMSO-d_6_) δ: 7.80 (d, 2H, J = 7.5Hz, CH-aromatic), 7.52 (dd, 2H, J = 7.4, 6.2 Hz, CH-aromatic), 7.41 (d, 2H, J = 8.3Hz CH-aromatic), 7.18 (d, 1H, J = 7.3Hz, CH-phenyl), 7.08 (s, 1H, CH-phenyl), 6.93 (d, 1H, J = 7.4Hz, CH-phenyl), 3.29 (s, 2H, CH_2_-benzylic); IR (KBr, cm^–1^): 3106, 3019, 1686, 1557, 984, 854; ^13^C-NMR (75 MHz, DMSO-d6) δ: 170.5, 143.4, 139.2, 133.8, 133.4, 131.8, 130.9, 129.5, 129, 128.6, 128.3, 128.1, 127.6, 127.1, 126.9, 126.5, 38.9; MS: m/z (%) 395 (M^+^, 46), 362 (13), 255 (53), 213 (100), 180 (85), 152 (54), 86 (83); Anal. Calcd for C_20_H_11_Cl_2_N_3_S: C, 60.62; H, 2.80; N, 10.60. Found: C, 60.53; H, 2.99; N, 10.44.

#### ***9-(4-chloro-benzylthio)-acenaphtho[1,2-e]-1,2,4-triazine (5e)***

Yield 84%, m.p. 153-156°C. ^1^HNMR (300 MHz, DMSO-d_6_) δ: 7.89 (d, 2H, J = 7.7Hz, CH-aromatic), 7.58 (dd, 2H, J = 7.5, 6.5 Hz, CH-aromatic), 7.42 (d, 2H, J = 8.6Hz CH-aromatic), 7.13 (d, 2H, J = 7.5Hz, CH-phenyl); 6.99 (d, 2H, J = 7.4Hz, CH-phenyl), 3.36 (s, 2H, CH_2_-benzylic); IR (KBr, cm^–1^): 3126, 3058, 1629, 1612, 1597, 1005, 917; ^13^C-NMR (75 MHz, DMSO-d6) δ: 169.9, 142.4, 138.7, 137.8, 132.7, 133.5, 129.2, 128.9, 128.3, 128, 127.7, 127.3, 126.1, 125.5, 38.5; MS: m/z (%) 361 (M^+^, 100), 328 (42), 208 (83), 180 (38), 164 (58), 125 (61); Anal. Calcd for C_20_H_12_ClN_3_S: C, 66.39; H, 3.34; N, 11.61. Found: C, 66.62; H, 3.38; N, 11.40.

#### ***9-(methylthio)-acenaphtho[1,2-e]-1,2,4-triazine (5f)***

Yield 31%, ^1^HNMR (500 MHz, CDCl_3_): 8.44-8.46 (m, 2H, CH-aromatic), 8.24 (m, 1H, CH-aromatic), 8.14 (d, 1H, J = 8.25 Hz, CH-aromatic), 7.86-7.90 (m, 2H, CH-aromatic), 2.84 (s, 3H, -CH_3_); IR (KBr, cm ^-1^): 3150, 2923, 1615, 1416 and 1382; Anal. Calcd for C_14_H_9_N_3_S: C, 66.91; H, 3.61; N, 16.72. Found: C, 66.82; H, 3.68; N, 16.59.

#### ***9-(ethylthio)-acenaphtho[1,2-e]-1,2,4-triazine (5g)***

Yield 30%, ^1^HNMR (500 MHz, DMSO-d_6_): 8.38-8.44 (m, 3H, CH-aromatic), 8.29 (d, 1H, J = 8.1 Hz, CH-aromatic), 7.91-7.96 (m, 2H, CH-aromatic), 3.33 (q, 2H, CH_2_-CH_3_), 1.44-1.47 (t, 3H, J = 7.2 Hz, CH_2_-CH_3_); IR (KBr, cm ^-1^): 3148, 2944, 1617, 1420 and 1381; Anal. Calcd for C_15_H_11_N_3_S: C, 67.90; H, 4.18; N, 15.84. Found: C, 68.06; H, 4.28; N, 15.73.

### Cytotoxicity assay

RPMI 1640, fetal bovine serum (FBS), trypsin and phosphate buffered saline (PBS) were purchased from Biosera (Ringmer, UK). 3-(4,5-dimethylthiazol-2-yl)-2,5-diphenyltetrazolium bromide (MTT) was obtained from Sigma(Saint Louis, MO, USA) and penicillin/streptomycin was purchased from Invitrogen (San Diego, CA, USA). Doxorubicin and dimethyl sulphoxide were obtained from EBEWE Pharma (Unterach, Austria) and Merck (Darmstadt, Germany), respectively.

HL-60 (human promyelocytic leukemia**)**, MCF-7 (human breast adenocarcinoma) and MOLT-4 (human acute lymphoblastic leukemia) cells were obtained from the National Cell Bank of Iran, Pasteur Institute, Tehran, Iran. All cell lines were maintained in RPMI 1640 supplemented with 10% FBS, and 100 units/mL penicillin-G and 100 μg/mL streptomycin. Cells were grown in monolayer cultures.

Cell viability following exposure to synthetic compounds was evaluated by using the MTT reduction assay. HL-60, MCF7, and MOLT-4 cells were plated in 96-well microplates at a density of 5 × 10^4^ cells⁄ mL (100 μl per well). Positive control wells contained cisplatin and doxorubicin and blank wells contained only growth medium for background correction. After overnight incubation at 37°C, half of the growth medium was removed and 50 μL of medium supplemented with different concentrations of synthetic compounds dissolved in DMSO were added in quadruplicate. Maximum concentration of DMSO in the wells was 0.5% (The solution of 0.5% DMSO was also tested as a cytotoxicity control). Cells were further incubated for 72 h. At the end of the incubation time, the medium was removed and MTT was added to each well at a final concentration of 0.5 mg⁄mL and plates were incubated for another 4 h at 37°C. Then formazan crystals were solubilized in 200 μl DMSO. The optical density was measured at 570 nm with background correction at 655 nm using a Bio-Rad microplate reader (Model 680). The percentage of viability compared to control wells was calculated for each concentration of the compound and IC50 values were calculated with the software CurveExpert version 1.34 for Windows. Each experiment was repeated 3–4 times. Data are represented as mean ± S.E.M.

### Molecular docking study

The ligand-flexible docking studies were performed using the widely distributed molecular docking software, AutoDock 4.2 [26]. Lamarckian Genetic Algorithm of the AutoDock 4.2 program was used to perform the flexible-ligand docking studies [27]. All the x-ray crystallographic *holo* structures of Bcl-2 were retrieved from the Brookhaven protein data bank (http://www.rcsb.org/). The protein structure was subjected to optimization step in order to minimize the crystallographic induced bond clashes using steepest descent method. All the preprocessing steps for receptor and ligand files were done by Auto-Dock Tools 1.5.4 (ADT) [28]. For the preparation of protein, Kollman united atom charges and polar hydrogen’s were added to the receptor and crystallographic waters were removed. For docked ligands, Gasteiger charge was assigned, non-polar hydrogens were merged into the related carbon atoms of the receptor and torsions degrees of freedom were also allocated by ADT program.

Lamarckian genetic algorithm (LGA) was used to simulate the binding affinity and binding mode of acenaphtho derivatives in the active site of Bcl-2. 100 independent genetic algorithm (GA) runs were considered for each ligand under study. For Lamarckian GA; 27000 maximum generations; a gene mutation rate of 0.02; and a crossover rate of 0.8 were applied. The grid maps of the protein were calculated using AutoGrid (part of the AutoDock package). The size of grid was set in a way to include not only the active site but also considerable portions of the surrounding surface. For this purpose, a grid of 60 × 60 × 60 points in x, y, and z directions was built centered on the center of mass of the catalytic site of Bcl-2 with a spacing of 0.375 Å. Cluster analysis was performed on the docked results using an Root mean square deviation (RMSD) tolerance of 2 Å.

Ligand-receptor interactions were all detected on the basis of docking results using LIGPLOT [29]. Molecular images were produced using VMD program [30].

## Results and discussion

### Chemistry

Some new acenaphtho derivatives were obtained by condensation of acenaphtylene-9,10-quinone and thiosemicarbazide followed by reaction with different benzyl chloride derivatives under mild conditions in chloroform solution (Scheme 1). The isolated compounds were then characterized by elemental analyses, FT-IR, MS and NMR spectroscopy. The applied synthetic method afforded all the 9-(alkylthio)-acenaphtho[1,2-e]-1,2,4-triazines in high yields and short reaction times except for 9-(methylthio) and 9-(ethylthio) derivatives that were produced in lower yields (Additional file 1: Table S1).

### Cytotoxicity assay

The *in vitro* cytotoxic activities for prepared acenaphtho derivatives are shown in Table 1.

**Table 1:** Cell growth inhibitory activity of synthetic acenaphtho derivatives assessed by the MTT reduction assay

**Comp. no.**	**IC**_**50**_^**a **^**(μM)**		
	**HL-60 cells**	**MCF-7 cells**	**MOLT-4 cells**
**5a**	48.4 ± 8.7	NA^b^	30.1 ± 5.6
**5b**	36.0 ± 5.4	NA	28.0 ± 4.6
**5c**	51.2 ± 7.6	NA	30.3 ± 8.2
**5d**	NA	NA	NA
**5e**	30.1 ± 3.6	NA	33.6 ± 2.9
**5f**	ND^c^	61.9 ± 20.6	65.5 ± 20.4
**5g**	ND	NA	NA
Cisplatin	3.0 ± 0.1	23.7 ± 6.8	3.0 ± 0.2
Doxorubicin	0.014 ± 0.002	0.221 ± 0.095	0.017 ± 0.002

^*a*^*Values represent mean ± S.E.M..*

^*b*^*NA: not active.*

^*c*^*ND: not determined.*

All the compounds under study exhibited medium cytotoxic activity on the evaluated cancerous cell lines except for 9-(methylthio) (5f) and 9-(ethylthio) (5 g) derivatives. Compound 5 g was inactive in MCF-7 and MOLT-4 cells. Compound 5b showed higher cytotoxicity in MOLT-4 cells. In HL-60 cells, compound 5e exhibited superior cytotoxic activity. The order of cytotoxic effects in HL-60 and MOLT-4 cell lines could be shown as 5e > 5b > 5a > 5c > 5d and 5b > 5a > 5c > 5e > 5f > 5d = 5 g. Variations of cytotoxic results were less pronounced for MOLT-4 cell lines. Moreover, all of the tested compounds were inactive on MCF-7 cells except for 5f which exhibited an IC_50_ value of 61.9 μM.

With the exception of compound **5e**, a common trend, might be deduced that the presence of electron withdrawing/hydrogen acceptor nitro group on the phenyl ring of 9-(alkylthio)-acenaphtho[1,2-e]-1,2,4-triazines afforded higher cytotoxic effects while halogenated compounds showed lower *in vitro* potencies. It was also revealed that acenaphtho derivatives bearing aralkyl substituents on their sulfur atom (5a-e) might exhibit better cytotoxicity profiles than acenaphtho derivatives bearing alkyl substituents (5f and 5 g). Further structure activity relationship developments is under investigation via preparing more diverse sets of these derivatives.

### Molecular modeling studies

We decided to gain some information on binding modes of tested compounds on the Bcl-2 active site via molecular docking. Previous reports proposed that despite the similarity in the structures of two Bcl-2 isoforms, they behave differently in binding to the pro-apoptotic members of the Bcl-2 family [31]. For this purpose we decided to conduct our modeling studies on the structures of the two isoforms of Bcl-2 protein (isoforms 1: 1G5M and isoform 2: 1GJH).

Due to the lower cytotoxic activities of 9-(methylthio) and 9-(ethylthio) derivatives and also considering our main focus on 9-(Aralkylthio)-acenaphtho[1,2-e]-1,2,4-triazines rather than 9-(alkylthio)-acenaphtho[1,2-e]-1,2,4-triazines, all modeling studies were performed on 9-(benzylthio) derivatives.

### Validation of molecular docking

A performance of a typical docking protocol can be checked via testing its ability in predicting predominant binding mode of a cognate (co-crystallographic) ligand. This procedure is performed via extracting the structure of a cognate ligand and re-docking it into its receptor (self-docking). RMSD of the Cartesian coordinates of the atoms of the ligand in the docked and crystallographic conformations will be the criterion of the docking validation (RMSD ≤ 2 Å).

Various PDB derived Bcl-2 structures were subjected to docking validation procedure. PDB structures were chosen on the basis of crystallographic resolutions. Regarding RMSD values and also conformation population in the top-ranked cluster of AutoDock output file, 1G5M and 1GJH were selected as the most appropriate crystallographic structure for further modeling studies.

### Docking simulation of acenaphtho derivatives

Considering the well obtained *in vitro* results, it was thought worthy to perform molecular docking studies, hence considering both *in silico* and *in vitro* results. Owing to the potential apoptosis-inducing activity of acenaphtho scaffolds via the Bcl-2 protein [17], we decided to model the binding interactions of assayed 9-(alkylthio)-acenaphtho[1,2-e]-1,2,4-triazines in the active site of Bcl-2 via docking simulation. For this purpose, 9-(alkylthio)-acenaphtho[1,2-e]-1,2,4-triazines were all docked into the active site of selected receptors *i.e.* various isoforms of Bcl-2 (1G5M and 1GJH) which differed in two amino acids [31].

Docking results with isoforms 1 (PDB code: 1G5M) and 2 (PDB code: 1GJH) are summarized in Tables 2 and 3, respectively. Top ranked binding energies (kcal/mol) in AutoDock dlg output files were considered as the best docking result in each case. It should be noted that all top ranked clusters were supported by high conformation populations. This observation could be expected since literature evidence implied that docking studies with compounds bearing less active torsions can significantly promote the docking success rates due to the limited conformational degrees of freedom [32].

**Table 2:** Top ranked AutoDock scores based on the binding free energies (ΔG) of acenaphtho structures docked into the Bcl-2 active site (Isoform 1: 1G5M) along with interacted amino acids of Bcl-2

**Comp. no.**	**ΔG**_**b **_**(kcal/mol)**	**H-bonds**		**Amino acids in non-bonded contacts**
		**Amino acid**	**Distance (Å)**	
**5a**	-7.03	Arg12	2.22	Glu13, Met16, Lys17, His20, Ala32, Asp35, Val36, Glu38, Asn39, Thr41
**5b**	-9.29	Glu48	1.95	Asp10, Gly46, Glu50
		Ser49	2.50	
		Lys17	1.73	
**5c**	-8.09	Arg12	1.96	Glu13, Met16, Ala32, Val36, Glu38, Asn39, Thr41
**5d**	-8.22	Arg12	2.20	Glu13, Met16, Ala32, Asp35, Val36, Glu38, Asn39, Thr41
**5e**	-7.73	Arg12	2.16	Glu13, Met16, Ala32, Asp35, Val36, Glu38, Asn39, Thr41

**Table 3:** Top ranked AutoDock scores based on the binding free energies (ΔG) of acenaphtho structures docked into the Bcl-2 active site (Isoform 2: 1GJH) along with interacted amino acids of Bcl-2

**Comp. no.**	**ΔG**_**b **_**(kcal/mol)**	**H-bonds**		**Amino acids in non-bonded contacts**
		**Amino acid**	**Distance (Å)**	
**5a**	-6.78	Ser49	2.18	Thr7, Pro44, Gly46, Glu48, Glu50
**5b**	-9.13	Asp10	1.87	Thr7, Gly8, Tyr9, Glu13, Thr41, Glu42, Ala43, Pro44, Gly46, Ser49
**5c**	-8.11	Ala32	2.36	Glu13, Met16, His20, Trp30, Asp31, Gly33, Val36
**5d**	-7.74	-		Glu13, Met16, His20, Glu29, Trp30, Ala32
**5e**	-7.84	-		Glu13, Met16, His20, Glu29, Trp30, Ala32

*In silico* results revealed that synthesized molecules showed relatively good binding energies toward Bcl-2 active site ranging from -7.03 to -9.29 kcal/mol in isoform1 (1G5M) and -6.78 to -9.13 kcal/mol in isoform 2 (1GJH). Estimated binding energies were in the order of 5b > 5d > 5c > 5e > 5a in the active site of isoform 1 and 5b > 5c > 5d > 5e > 5a in the active site of isoform 2. The trends may also highlight slight conformational differences for two Bcl-2 isoforms [31].

**Figure 1:**
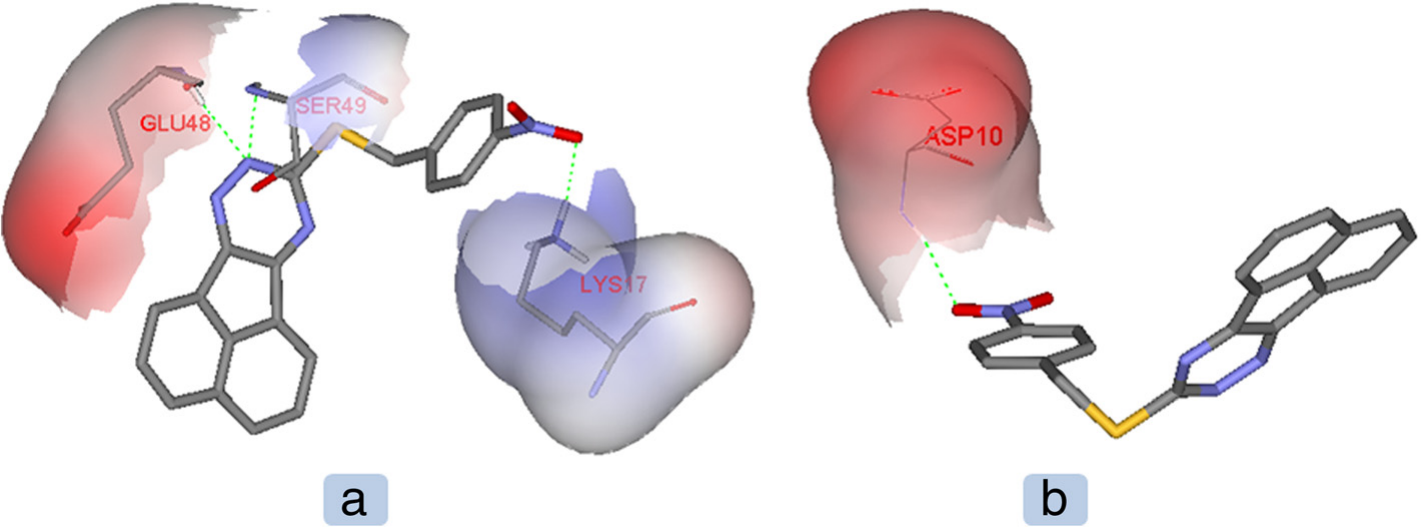
H-bond interactions of compound 5b in the active site of Bcl-2 isoforms a) PDB ID: 1G5M and b) PDB ID: 1GJH.

**Figure 2:**
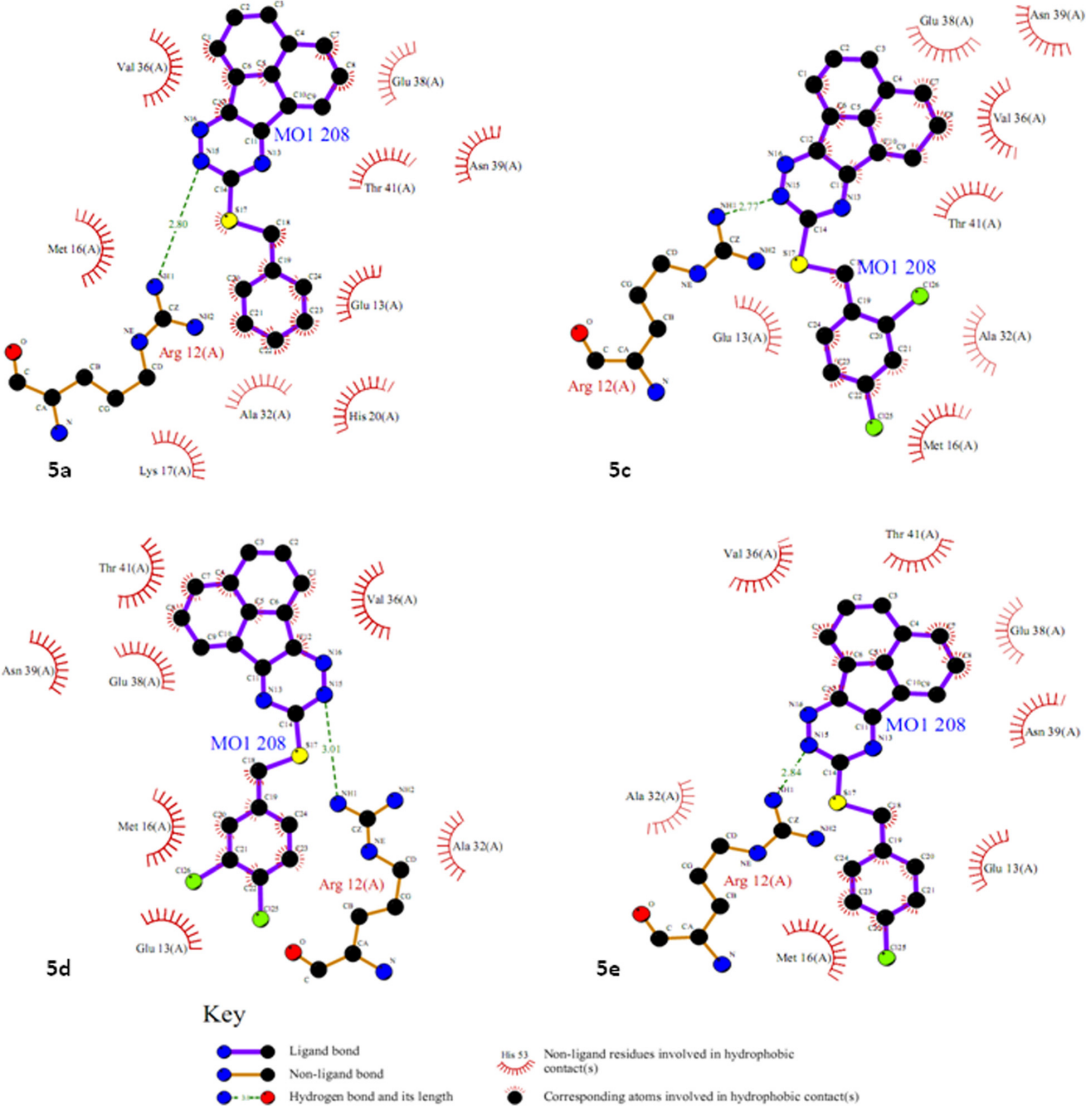
H-bond interactions between acenaphtho derivatives (5a, 5c, 5d and 5e) and active site residues in isoform 1 of Bcl-2.

**Figure 3:**
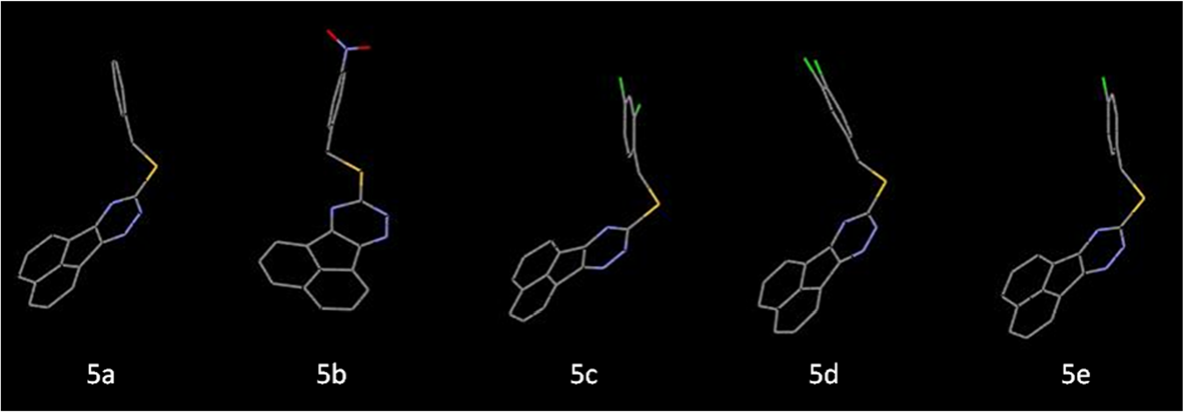
Different active site oriented conformations for docked acenaphtho derivatives (5a, 5b, 5c, 5d and 5e) in the active site of Bcl-2 isoform 1.

Molecular docking study revealed that 9-(alkylthio)-acenaphtho[1,2-e]-1,2,4-triazine derivatives (5a-e) may inhibit Bcl-2 protein via H-bond and hydrophobic interactions (Tables 2 and 3). Compound 5b exhibited the best docking score (Tables 2 and 3) while being most potent in human MOLT-4 cells (Table 1). It seemed that the presence of *para*-nitro substituent on phenyl ring of 5b might be responsible for additional H-bonds with the active site of the receptor. This H-bond acceptor site would potentially support one key H-bond interactions with NH of Lys17 in isoform 1 and NH of Asp10 in isoform 2 through oxygen atom of a nitro substituent (Figure 1). The profile of interaction in 5b (Figure 1) further announced us that *para* nitro substituent in *9-(4-nitro-alkylthio)-acenaphtho[1,2-e]-1,2,4-triazine* (5b) might have some directional effect on the active site oriented conformation of the ligand. This rationale could be shown in Figure 2 in which different orientation of acenaphtho cycle in 5b compared to other compounds is obviously detectable. This new orientation would relocate a triazine ring in a position to make H-bond interactions with Ser49 and Glu48 via polar hydrogen of N15 atom (Figures 1 and 3). From another aspect of view, docking simulation could be applied to find the binding mode and mechanism of less active derivatives.

To add more, it seemed that H-bond connections contributed more significantly in complex stabilization in the case of 5b and Bcl-2 isoform 1 (1G5M) rather than isoform 2 (1GJH). Compounds 5a, 5c, 5d and 5e all participated in key H-bonds with nitrogen atoms of a triazine ring **(**Figure 2). We found that Arg12 interacted via its guanine side chain (NH1; Figure 2) with nitrogen atom of a triazine ring in acenaphtho derivatives (N15; Figure 2).

Docking of commonly used neoplastic drugs *i.e.* Geftinib, Cisplatin, 5-FU, Gemcitabine and Vinorelbine into the active site of Bcl-2 (isoform 1; 15GM) has been reported elsewhere [6]. It was revealed that Asp10, Glu13, Lys17, Glu42 and Ser49 contributed to H-bond formation with the docked drugs [6]. Same residues were found to be key H-bond/non-bonded participants for inhibitor recognition in our study. Docking results showed that Glu13 contributed to non-bonded contacts with 5a, 5b, 5c and 5d (Table 1) while Asp10 participated in non-bonded contacts with 5b. Moreover; H-bond network in the case of Cisplatin (Asp10, Lys17 and Ser49) was very similar to that of 5b (Lys17, Glu48 and Ser49). The residue atoms involved in the binding pattern were the same for cisplatin and 5b in the case of Lys17 (quaternary NH of side chain; Figure 1) while for Ser49, cisplatin interacted with hydroxyl group of the Ser49 side chain but compound 5b made hydrogen bond via Ser49 backbone NH (Figure 1). Lys17 has also been reported to be involved in efficient H-bonds with Geftinib, Gemcitabine and Vinorelbine while stabilizing H-bonds were detected in the case of Glu13 for Gemcitabine and Vinorelbine [6].

**Figure 4:**
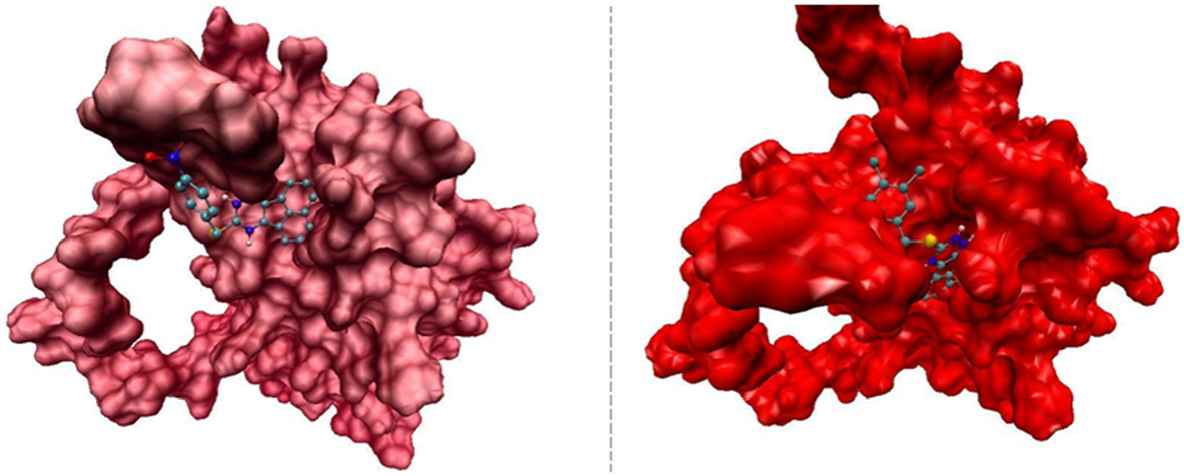
Compounds 5b (left) and 5d (right) in the active site of Bcl-2.

Literature data revealed the effect of position of substituent(s) on phenyl moiety in comparison to our results [33]. It has been discussed that considering substituent groups being attached to the *para* or *meta*-position, the hydrophobic pocket of Bcl-2 exhibited varied tolerability. This further identified the size of the hydrophobic pocket of Bcl-2 to be limited. *Meta* or *para*-halogen atoms on phenyl ring induce different conformations leading to decreased interactions with hydrophobic pocket of Bcl-2 (compounds 5d and 5f) (Figure 4).

Molecular docking on human anti-apoptotic Bcl-2 further supported the biological data. Regarding the obtained results, compound 5b could serve as an appropriate starting point for designing new chemical entities as potent Bcl-2 inhibitors.

### Binding efficiency indices

Another criterion which has recently absorbed much attention in ligand-receptor interaction studies is the ligand efficiency (LE) parameter. Nowadays LE indices are regarded as undeniable tools in modern drug discovery projects [34]. The model of analyzing ligand binding in terms of the free energy per heavy atom (heavy atom count; HAC) was first introduced by Andrews [35]. Concept of the binding energy per atom or binding efficiency of a ligand could be a useful parameter in the selection of lead compounds, considering the real potency of a compound and hence might consider optimized fragments [36]. Generally speaking, molecules achieving a desirable potency with fewer HACs are by definition more efficient. LE index can be simply calculated using the equations (1) or (2):

(1) $$\mathrm{LE}=‒\frac{\Delta G_{b}}{\text{HAC}}$$

(2) $$\mathrm{LE}=‒\frac{pK_{i};pK_{d;}\mathrm{or}\;\text{pIC}50}{\text{HAC}}$$

Regarding the ligand efficiencies, it was postulated that molecular weights are prior to the HACs in considering the contribution of heteroatoms from different rows of the periodic table [37]. Thus a modified efficiency value was suggested as binding efficiency index (BEI). The importance of BEI can be emphasized regarding an increase in molecular weight at the clinical candidate step, which is regarded as an undeniable paradox with a common trend towards lower MWs and better pharmacokinetic profiles in marketable drugs [38]. BEI could be easily estimated from equation (3):

(3) $$\text{BEI}=‒\frac{pK_{i};\;\mathrm{or}\;\text{pIC}50}{\mathrm{MW}\;\left(\text{kDa}\right)}$$

Accordingly, assayed acenaphtho derivatives were re-evaluated on the basis of their experimental and theoretical BEI/LE values (Table 4).

**Table 4:** **Experimental/theoretical binding/ligand efficiency indices for 9-(benzylthio)-acenaphtho[1,2-e]-1,2,4-triazine derivatives (Bcl-2, isoforms 1** & **2, PDB IDs: 1G5M** & **1GJH)**

**HL-60 cells**							**MOLT-4 cells**								
**Molecule no.**	**Related PDB code**	**IC50 (μM)**	**pIC50**	**Experimental LE**^**a**^	**Estimated LE**^**b **^**(kcal.mol**^**-1**^**.HAC**^**-1**^**)**	**Experimental BEI **^**c**^	**Estimated BEI (kcal.mol**^**-1**^**.kDa**^**-1**^**)**	**Molecule no.**	**Related PDB code**	**IC50 (μM)**	**pIC50**	**Experimental LE**	**Estimated LE (kcal.mol**^**-1**^**.HAC**^**-1**^**)**	**Experimental BEI**	**Estimated BEI (kcal.mol**^**-1**^**.kDa**^**-1**^**)**
**5a**	1G5M	48.4	4.32	0.179	0.208	13.20	15.77	**5a**	1G5M	30.1	4.52	0.188	0.208	13.83	15.77
**5b**	1G5M	36.0	4.44	0.114	0.134	11.95	14.36	**5b**	1G5M	28.0	4.55	0.190	0.134	12.24	14.36
**5c**	1G5M	51.2	4.29	0.165	0.228	10.86	15.01	**5c**	1G5M	30.3	4.52	0.188	0.228	11.44	15.01
**5d**	1G5M	-	-	-	0.218	-	15.26	**5d**	1G5M	-	-	-	0.218	-	15.26
**5e**	1G5M	30.1	4.52	0.170	0.219	12.52	15.69	**5e**	1G5M	33.6	4.47	0.186	0.219	12.39	15.69
**5a**	1GJH	48.4	4.31	0.179	0.214	13.20	15.24	**5a**	1GJH	30.1	4.52	0.188	0.214	13.83	15.24
**5b**	1GJH	36.0	4.44	0.114	0.137	11.95	14.05	**5b**	1GJH	28.0	4.55	0.190	0.137	12.24	14.05
**5c**	1GJH	51.2	4.29	0.165	0.228	10.86	15.04	**5c**	1GJH	30.3	4.52	0.188	0.228	11.44	15.04
**5d**	1GJH	-	-	-	0.232	-	14.36	**5d**	1GJH	-	-	-	0.232	-	14.36
**5e**	1GJH	30.1	4.52	0.170	0.237	12.52	15.18	**5e**	1GJH	33.6	4.47	0.186	0.237	12.39	15.18

^*a*^*LE values are defined in the range of 0–1 *[34]*.*

^*b*^*Estimated LEs were calculated according to equation (2).*

^*c*^*Reference value for BEI is 27 *[35]*.*

Regarding the summarized data in Table 4, following rationales might be pointed out:

– In our calculations, estimated LEs in terms of pKi values (equation 2) produced results more compatible to the experimental LEs (cellular assays) when compared to the LEs in terms of ΔG_b_ values (equation 1; the related data are not shown in the Table).

– Compound 5b is the top ranked bioactive molecule in docking results and also MOLT-4 based cellular assay. But this compound achieved relatively lower scores in terms of experimental/theoretical efficiency indices. The observed paradox demonstrated that larger bioactive compounds may require special attention in their design toward efficiency indices [25].

– Efficient bioactive molecules may be better categorized considering estimated BEIs rather than LEs. In some cases, especially for molecules bearing larger atoms, incorporating molecular weights rather than number of heavy atoms in the calculation of efficiency indices produced less biased results.

– The application of theoretical ligand/binding efficiency indices in the cell –based cytotoxicity protocols should be considered with care due to the multi-target nature of the cytotoxic agents. For example, compound 5d which is the structural isomer of compound 5c, was inactive in MTT assays.

## Conclusion

Various studies have demonstrated that chemical structures bearing sulfur atom(s) may possibly induce apoptosis [39,40]. On the basis of results from this study, corresponding 9-(benzylthio) acenaphtho[1,2-e]-1,2,4-triazines might be regarded as valuable cytotoxic polycyclic heterocycles and potential candidates for additional molecular modifications with the aim of developing potent cytotoxic agents. Experimental and modeling studies confirmed the previous reports on acenaphtho derivatives and moreover; the docking results agreed well with the observed *in silico* data for commonly used anticancer drugs. Despite the closeness of docking scores, relatively different binding profiles for acenaphtho derivatives might confirm the previous results on conformational difference between two Bcl-2 isoforms. Concept of binding efficiency as a common and well–approached tool in modern drug discovery strategies was reviewed in our study. Confirming previous reports, it was demonstrated that bioactive molecular design on the basis of potency alone or binding efficiency values might be considered as different strategies leading to dissimilar results in lead/drug development campaigns. However several literature reports demonstrated that lead/drug discovery projects on the basis of binding efficiency indices would afford bioactive compounds with better pharmacokinetic outcomes. Extended cytotoxicity assays on diverse sets of acenaphtho-based scaffolds with the aim of establishing more rational structure activity relationships are under investigation.

## Competing interests

The authors declare that they have no competing interests.

## Authors’ contributions

MKM: Synthesis of some target compounds. OF: Supervision of biological tests. MKH: Design of target compounds and collaboration in manuscript preparation. NR-A: Performed the molecular docking study and collaboration in manuscript preparation. SS: Performed the molecular docking study. RM: supervision of the design, synthetic and pharmacological parts. All authors read and approved the final manuscript.

## Supplementary Material

Additional file 1: Table S1 Physical and analytical data of 9-(alkylthio)-acenaphtho[1,2-e]-1,2,4-triazines 5a-h. Click here for file
