# Supplementary material for: Novel 9-(alkylthio)-Acenaphtho[1,2-e]-1,2,4-triazine derivatives: synthesis, cytotoxic activity and molecular docking studies on B-cell lymphoma 2 (Bcl-2)
Source: Daru. 2014 Jan 6;22(1):2. doi: 10.1186/2008-2231-22-2 (PMC3917598; doi:10.1186/2008-2231-22-2)
Supplement: Additional file 1: Table S1 — Physical and analytical data of 9-(alkylthio)-acenaphtho[1,2-e]-1,2,4-triazines 5a-h. [file 2008-2231-22-2-S1.doc]

**Table 1** **Physical and analytical data of 9-(alkylthio)-acenaphtho[1,2-e]-1,2,4-triazines 5a-h**

**
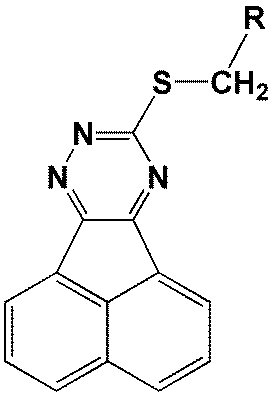
**

| **m.p (°C)** | **Yield** | **Time** | **Molecular formula** | **R** | **Comp. No.** |
| --- | --- | --- | --- | --- | --- |
|  | **(%)** | **(min)** | **(Mol. Wt.)** |  |  |
| 151-153 | 94 | 40 | C_20_H_13_N_3_S  327.08 | 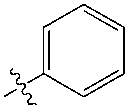 | 5a |
| 156-158 | 79 | 50 | C_20_H_12_N_4_O_2_S  372.07 | 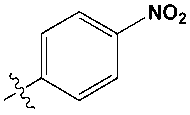 | 5b |
| 187-188 | 83 | 75 | C_20_H_11_Cl_2_N_3_S  395.01 | 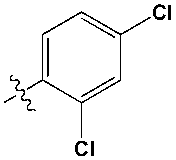 | 5c |
| 156-158 | 60 | 52 | C_20_H_11_Cl_2_N_3_S  395.01 | 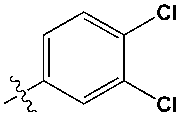 | 5d |
| 153-156 | 84 | 82 | C_20_H_12_ClN_3_S  361.04 | 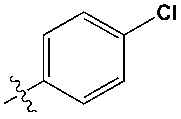 | 5e |
| - | 31 | 24 | C_14_H_9_N_3_S  251.31 | 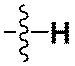 | 5f |
| - | 30 | 24 | C_15_H_11_N_3_S  265.33 | 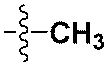 | 5g |
